# Ill-Health Amongst Plymouth School Children

**Published:** 1917-05-26

**Authors:** 


### Ill-health Amongst Plymouth School Children.

As one result of medical inspection in the Plymouth elementary schools a report has been presented on "exceptional" children by the Secretary of Education, Mr. Chandler Cook. This is a document of more than passing interest. It reveals the astonishingly large total of 2,603 "exceptional" children (1,183 boys and 1,420 girls) in the Plymouth schools. Moreover, it does not profess to be a complete return, and in regard to tuberculosis cases the figure must be taken as the lowest computation. The detailed analysis shows that of the scholars attending the primary schools of the town the following are reported as suffering from either defects in health, body, or mind:—

| | Boys | Girls |
|---|---|---|
| Blind or partially blind ... ... | 54 | 19 |
| Deaf or deaf and dumb ... ... | 68 | 84 |
| Feeble-minded ... ... ... | 237 | 192 |
| Epileptics ... ... ... ... | 41 | 33 |
| Deformed (cripples, paralytics, spinal cases) ... ... ... | 94 | 79 |
| Pulmonary tuberculosis ... ... | 111 | 146 |
| Non-pulmonary tuberculosis ... | 38 | 38 |
| Dull or backward ... ... ... | 540 | 829 |
| | 1,183 | 1,420 |

Of these 2,603 "exceptional" children, only 436 are attending special schools, and it is now urged that provision shall be made for the training and treatment of the others at two new residential open-air schools and at an experimental vacation school. The education authority's scheme to remedy this waste of young life includes, of course, hospital treatment for some.

